# Predictive Factors for Clinical Outcome After Direct Mechanical Thrombectomy for Anterior Circulation Large Vessel Occlusion Within 4.5 h

**DOI:** 10.3389/fneur.2022.895182

**Published:** 2022-06-30

**Authors:** Huu An Nguyen, Dang Luu Vu, Quang Anh Nguyen, Duy Ton Mai, Anh Tuan Tran, Hoang Kien Le, Tat Thien Nguyen, Thu Trang Nguyen, Cuong Tran, Viet Phuong Dao, Laurent Pierot

**Affiliations:** ^1^Department of Radiology, Hanoi Medical University, Hanoi, Vietnam; ^2^Radiology Center, Bach Mai Hospital, Hanoi, Vietnam; ^3^Stroke Center, Bach Mai Hospital, Hanoi, Vietnam; ^4^Department of Neuroradiology, Hôpital Maison-Blanche, Université Reims-Champagne-Ardenne, Reims, France

**Keywords:** direct mechanical thrombectomy, acute ischemic stroke, anterior circulation, large vessel occlusion, efficacy, prognostic factors, clinical outcome

## Abstract

**Background:**

Recent trials including DIRECT-MT, DEVT, and SKIP have found that direct mechanical thrombectomy (MT) is equally effective as the combination of MT and intravenous thrombolysis. However, the results of the other trials, namely MR-CLEAN NO-IV and the SWIFT-DIRECT trial have failed to confirm the non-inferiority of direct MT vs. the combination therapy.

**Aim:**

We aimed to identify prognostic factors of direct MT for anterior circulation large vessel occlusion within 4.5 h.

**Materials and Methods:**

Data from January 2018 to January 2022 were retrospectively collected and analyzed. Adult patients with confirmed anterior circulation large vessel occlusion within 4.5 h of onset with baseline NIHSS of ≥6 and baseline ASPECTS of ≥6 treated using direct MT within 6 h were recruited.

**Results:**

A total of 140 patients were enrolled in the study with a median age of 65.5 years [interquartile range (IQR), 59–76.5], median baseline NIHSS of 13.5 (IQR, 11–16), and median baseline ASPECTS of 8 (IQR, 7–8). Direct MT was feasible in all patients (100%). Successful reperfusion (mTICI 2b-3) was achieved in 124/140 patients (88.6%) with a low rate of complications (8/140, 5.7%). Any type of intracranial hemorrhage (ICH) and symptomatic ICH occurred in 44/140 (31.4%) and 5/140 (3.6%), respectively. Overall, a good outcome (mRS 0–2) was achieved in 93/140 (66.4%), and the mortality rate was 9.3% (13/140 patients). Using multivariate analysis, lower age [odds ratio (OR), 0.96; 95% CI, 0.92–1.00; *P* = 0.05], low baseline NIHSS (OR, 0.82; 95% CI, 0.74–0.92; *P* = 0.00), and absence of ICH (OR, 0.29; 95% CI, 0.10–0.81; *P* = 0.02) were independently associated with favorable outcome. Independent predictors of mortality were baseline NIHSS (OR, 1.21; 95% CI, 1.01–1.46; *P* = 0.04), successful reperfusion (OR, 0.02; 95% CI, 0.00–0.58; *P* = 0.02), and ICH (OR, 0.12; 95% CI, 0.02–0.75; *P* = 0.02). Further analysis showed that the median mRS at 90 days was significantly better in the MCA occlusion group compared to the ICA plus M1 occlusion group [1 (IQR 0–3) vs. 2 (IQR 1–4); *P* = 0.05].

**Conclusions:**

Our findings suggest that direct thrombectomy may be an adequate clinical option for younger patients (≤70) experiencing proximal middle artery occlusion within 4.5 h and who have low baseline NIHSS (≤14).

## Introduction

Acute stroke is a major cause of mortality worldwide, accounting for 11% of all-cause mortality (1). Acute ischemic stroke (AIS) is the primary disease type and contributes up to 85% of all strokes (2). Stroke survivors not only have a high risk of death but can also suffer severe sequelae or even lifelong disability. Therefore, in the last three decades, remarkable efforts in medical research have focused on understanding AIS preventive factors as well as developing new treatments.

The first landmark in AIS treatment was the success of the Tissue Plasminogen Activator for Acute Ischemic Stroke trial on the efficacy of intravenous thrombolysis (IVT) with alteplase in 1995 (3). For nearly 20 years, IVT has been the sole treatment for AIS within 4.5 h of onset. However, this therapy has two major drawbacks: low recanalization rate in large vessel occlusion (LVO) (4) and increased risk of intracranial hemorrhage (ICH) (3). Many attempts to develop and test newer generation thrombolytic agents have also been conducted in the past few years, prompting the introduction of tenecteplase, which is considered to be more active than alteplase (5).

The second landmark was the success of five randomized controlled trials (RCTs) in 2015 of mechanical thrombectomy (MT) for patients with LVO (6–10). This method has been shown to provide better functional outcomes than IVT alone in AIS due to LVO of the anterior circulation (11). Since then, the treatment paradigm for AIS caused by LVO has shifted to MT taking the central role.

The 2019 Guidelines of the American and European Stroke Associations recommended that IVT be used in parallel with MT for eligible patients with AIS due to LVO within 4.5 h (12, 13). However, this approach is controversial due to the relatively low recanalization rate of IVT before MT [only 7.6% in the Endovascular thrombectomy after large-vessel ischemic stroke: a meta-analysis of individual patient data from five randomized trials (11)] and the increased risk of ICH (14). Moreover, a meta-analysis of pivotal MT trials found no significant differences in the safety and efficacy between direct MT and the combination of MT and IVT (15). Therefore, there has been a global debate regarding the requirement for IVT before MT for patients experiencing AC-LVO within 4.5 h and who are admitted to hospitals capable of performing direct MT. Three RCTs comparing direct MT with the combination therapy including Endovascular Thrombectomy with or without Intravenous Alteplase in Acute Stroke (DIRECT-MT), Effect of Endovascular Treatment Alone vs. Intravenous Alteplase Plus Endovascular Treatment on Functional Independence in Patients With Acute Ischemic Stroke (DEVT), and Effect of Mechanical Thrombectomy Without vs. With Intravenous Thrombolysis on Functional Outcome Among Patients With Acute Ischemic Stroke (SKIP) have confirmed the non-inferiority of direct MT vs. the combination therapy for AIS caused by AC-LVO within 4.5 h (16–18). However, results from other RCTs including A Randomized Trial of Intravenous Alteplase before Endovascular Treatment for Stroke (MR CLEAN-NO IV) and Solitaire™ With the Intention For Thrombectomy Plus Intravenous t-PA vs. DIRECT Solitaire™ Stent-retriever Thrombectomy in Acute Anterior Circulation Stroke (SWIFT-DIRECT) have failed to show either superiority or non-inferiority of direct MT over combination therapy (19, 20).

More recently, the latest guidelines from European Stroke Organization (ESO)-European Society for Minimally Invasive Neurological Therapy (ESMINT) recommended using IVT plus MT over MT alone for stroke patients with AC-LVO within 4.5 h (21). However, there is still a need for finding out which individuals' direct MT would indeed be non-inferior to combination therapy (16–18). In the context of the current post-COVID economic crisis, the shortage of resources even in health care has occurred in some countries, especially in developing countries. It is necessary to develop personalized medicine to answer the key question of how to best allocate resources so that the right patient can receive the right therapeutic strategy to eliminate futile therapies. With this in mind, we tried to provide further clinical evidence to identify predictive factors for outcomes of direct MT strategy.

## Materials and Methods

### Study Design

This descriptive study was conducted at Bach Mai Hospital, the largest and most comprehensive stroke center in northern Vietnam. Approximately 10,000 episodes of stroke and 300 MT procedures occur in our hospital each year. Patients with AIS caused by anterior circulation large vessel occlusion (AC-LVO) within 4.5 h who had relative contraindications of IVT and were treated with direct MT from January 2018 to April 2020 were retrospectively enrolled. Additionally, with the approval of the Independent Ethics Committee of Vietnam's Ministry of Health (No. 35/CN-HÐÐÐ), from April 22, 2020, several patients without contraindications of IVT were recruited in an interventional trial comparing direct MT with combination therapy. Data from all these patients have been permitted to use by the patient or their representative.

Inclusion criteria were as follows: (1) written informed consent; (2) age ≥ 18; (3) AIS with National Institutes of Health Stroke Scale (NIHSS) ≥ 6; (4) causative occlusion of the internal carotid artery (ICA) or proximal middle cerebral artery segments (M1, M2) within 4.5 h of symptom onset; (5) Alberta Stroke Program Early CT Score (ASPECTS) ≥ 6; (6) CT or MRI ruling out ICH; and (7) groin puncture for MT within 6 h of symptom onset. Exclusion criteria included (1) the use of any dose of IVT; (2) pre-stroke modified Rankin Scale (mRS) >2; (3) missing 3-month mRS score; and (4) refusal to participate in the study.

Clinical variables recorded were age, gender, background medical conditions (previous stroke, atrial fibrillation, hypertension, diabetes mellitus), and baseline NIHSS. Imaging variables were baseline ASPECTS on non-enhanced CT (NECT) or diffusion-weighted imaging (DWI) and occlusion site (ICA, M1, M2, or tandem occlusion) on CTA or MRA. Procedural variables were time from onset to groin puncture, procedure time, time from onset to reperfusion, type of anesthesia, number of passages, use of rescue therapy, and complications (perforation/dissection). Outcome variables were reperfusion results at procedure end according to the modified treatment in cerebral infarction (mTICI) scale (22), the presence of ICH after treatment, and functional outcome at 3 months. Successful reperfusion was defined as mTICI grade 2b or 3. The first-pass reperfusion was successful recanalization (mTICI 2b-3) after a single thrombectomy pass. Symptomatic ICH (sICH) was defined using the Safe Implementation of Thrombolysis in Stroke-Monitoring Study (SITS-MOST) criteria (23). Good outcome was defined as mRS between 0 and 2 at a 3-month follow-up.

### Mechanical Thrombectomy

Endovascular therapy was performed by a senior neurointerventionalist (>4 years of experience) and a junior doctor under a DSA monoplane (Philips Allura Xper FD20) or a DSA biplane (Philips Allura Xper FD10/10). The type of anesthesia (conscious sedation or general anesthesia) was determined according to the clinical status of the patient and the preference of each interventionist according to the 2018 guideline from the American Stroke Association (12). To locate the occlusion site, a large long-sheath (Neuron Max 088, Penumbra, Alameda, CA, USA) was placed in the carotid artery for angiography. Thrombectomy was performed with a second-generation stent retriever (Solitaire, Medtronic, Irvine, CA, USA; Trevor, Stryker Neurovascular, Mountain View, CA, USA) or aspiration tubing (Sofia Plus catheter, MicroVention, CA, USA; ACE or Jet 7 catheter, Penumbra, Alameda, CA, USA; React catheter, Medtronic), or combined technique at the neurointerventist's discretion. Rescue therapies including balloon angioplasty, extra-cranial, or intra-cranial stenting were performed in the event of LVO due to atherosclerosis disease, with the potential addition of antiplatelet agents. The procedure was completed with the use of an Angio-Seal 8F (Terumo Interventional Systems, Somerset, NJ, USA) device or manual compression for femoral artery closure.

### Outcome Measurement

Reperfusion results were graded on the final angiogram using the mTICI scale. ICH was confirmed on the NECT or T2^*^ sequence of MRI from 18 to 30 h post-intervention. NIHSS was also re-evaluated to determine if it was sICH. Functional outcomes were assessed by the mRS at 3-months post-treatment at a planned visit or *via* telephone.

### Statistical Analysis

Continuous variables are presented as medians and interquartile ranges (IQR) or mean and standard deviation (SD). Categorical variables are presented as percentages.

Clinical, imaging, procedural, and outcome variables were compared using the Mann-Whitney U test for continuous variables and the Fisher exact test for categorical variables. Univariate and multivariate logistic regression were analyzed to identify the odds ratio (OR) for any association between prognostic factors and outcomes. *P*-value ≤ 0.05 was accepted as a statistically significant difference for a 95% confidence interval (CI). Data were processed and analyzed using SPSS software (version 16.0; IBM SPSS Inc., Chicago, IL, USA).

## Results

From January 2018 to January 2022, 140 consecutive patients presenting with AC-LVO within 4.5 h and treated with direct MT were enrolled in the study. From April 2020, 45 patients without contraindications of IVT were recruited for an interventional trial comparing direct MT vs. the combination therapy, and the remaining 95 patients with relative contraindications of IVT were retrospectively enrolled from our stroke registry. Relative contraindications of IVT in these 95 patients included oral anticoagulant uses (60 patients), advanced age (older than 80 years) (18 patients), early ischemic changes more than one-third of the MCA territory (ASPECTS < 7) (11 patients), the combination of both previous ischemic stroke and diabetes mellitus (4 patients), severe stroke (NIHSS > 25) (2 patients).

### Study Population and Outcomes

The clinical, imaging, procedural characteristics, and outcomes of the patient cohort are shown in Table 1. Seventy-three women (52.1%) and 67 men (47.9%) with a median age of 65.5 years (IQR, 59–76.5) were enrolled. The median baseline NIHSS was 13.5 (IQR, 11–16). Hypertension (75 patients, 53.6%) and atrial fibrillation (44 patients, 31.4%) were the most common identified risk factors for stroke in this cohort.

**Table 1:** Patient characteristics and outcomes (*n* = 140).

**Clinical characteristics**	
Age, year, median (IQR)	65.5 (IQR, 59–76.5)
Gender, male, *n* (%)	67 (47.9%)
Baseline NIHSS, median (IQR)	13.5 (IQR, 11–16)
Previous stroke, *n* (%)	24 (17.1%)
Atrial fibrillation, *n* (%)	44 (31.4%)
Hypertension, *n* (%)	75 (53.6%)
Diabetes mellitus, *n* (%)	7 (5%)
**Imaging characteristics**	
Pure ICA occlusion, *n* (%)	8 (5.7%)
ICA plus M1 occlusion, *n* (%)	31 (22.2%)
M1 occlusion, *n* (%)	71 (50.7%)
M2 occlusion, *n* (%)	17 (12.1%)
Tandem occlusion, *n* (%)	13 (9.3%)
Baseline ASPECTS, median (IQR)	8 (IQR, 7–8)
**Procedural characteristics**	
General anesthesia, *n* (%)	116 (82.9%)
Time to start of treatment, minute, median (IQR)	250 (IQR, 190–274)
Procedure time, minute, median (IQR)	32.5 (IQR, 19.3–60.8)
Time to reperfusion, minute, median (IQR)	291.5 (IQR, 230–337)
Stent retriever first, *n* (%)	44 (31.4%)
Aspiration tubing first, *n* (%)	46 (32.9%)
Combined technique first, *n* (%)	50 (35.7%)
Number of passages, median (IQR)	1 (IQR, 1–2)
First-pass reperfusion (mTICI 2b-3), *n* (%)	69 (49.3%)
Rescue therapy, *n* (%)	10 (7.1%)
Perforated complication, *n* (%)	8 (5.7%)
**Outcome characteristics**	
Successful reperfusion (mTICI 2b-3), *n* (%)	124 (88.6%)
Any intracranial hemorrhage, *n* (%)	44 (31.4%)
Symptomatic intracranial hemorrhage, *n* (%)	5 (3.6%)
mRS (0–2) at 3 months, *n* (%)	93 (66.4%)
mRS (3–6) at 3 months, *n* (%)	47 (33.6%)
Mortality, *n* (%)	13 (9.3%)

Of these 140 patients, 39 (27.9%) had occlusions in the ICA (pure ICA occlusion in 8 patients and ICA plus M1 occlusion in 31 patients), 71 (50.7%) in the M1, 17 (12.1%) in the M2, and the remaining 13 (9.3%) had tandem occlusion. The median baseline ASPECTS was 8 (IQR, 7–8) and the majority (128/140 patients, 91.4%) had ASPECTS ≥ 7.

General anesthesia was used in the majority of procedures (116/140 patients, 82.9%). MT was feasible in all patients (100%). The median time from stroke onset to groin puncture was 250 min (IQR, 190–274); the median procedure time from groin puncture to reperfusion was 32.5 min (IQR, 19.3–60.8); and the median time from stroke onset to reperfusion was 291.5 min (IQR, 230–337). About the first thrombectomy device, a stent retriever was used in 44 patients (31.4%), aspiration tubing was used in 46 patients (32.9%), and the combined technique was used in 50 patients (35.7%). The median number of passages was 1 (IQR, 1–2), and first-pass reperfusion was achieved in 69/140 patients (49.3%). Rescue techniques were used in 10/140 patients (7.1%).

Overall, successful reperfusion (mTICI 2b-3) was achieved in 124/140 patients (88.6%). Arterial perforation resulting in subarachnoid hemorrhage occurred in 8/140 patients (5.7%). During post-treatment follow-up, any type of ICH occurred in 44/140 patients (31.4%), of which 5 patients (3.6%) were sICH. Good outcome (mRS 0–2) was achieved in 93/140 patients (66.4%) with a mortality rate of 9.3% (13/140).

### Prognostic Factors for Favorable Outcome

Table 2 shows the statistically significant differences in age, baseline NIHSS, successful reperfusion rate, and rate of any ICH based on the outcome. In univariate analysis, all four variables were identified as associated with good outcomes at 3 months. However, in multivariate analysis, only younger patients (OR, 0.96; 95% CI, 0.92–1.00, *P* = 0.00), low baseline NIHSS (OR, 0.82; 95% CI, 0.74–0.92; *P* = 0.00), and absence of any ICH (OR, 0.29; 95% CI, 0.10–0.81; *P* = 0.02) were identified as independently associated with good outcome at 3 months, as shown in Table 3. When dichotomized, patients aged ≤ 70 years (73.6 vs. 53.1%; OR, 2.47; 95% CI, 1.19–5.12; *P* = 0.02) and those with baseline NIHSS ≤ 14 (76.1 vs. 47.9%; OR, 0.29; 95% CI, 0.14–0.61; *P* = 0.00) had better outcomes.

**Table 2:** Factors associated with favorable outcome based on modified Rankin Score.

**Outcome**	**mRS 0-2** **(*n* = 93)**	**mRS 3-6** **(*n* = 47)**	***P-*value**
Age, year, median (IQR)	65 (56–73)	69 (60–80)	0.03^*^
Gender, male, *n* (%)	43 (46.2%)	24 (51.1%)	0.59
Baseline NIHSS, median (IQR)	12 (10–14.5)	15 (12–20)	0.00^*^
Previous stroke, *n* (%)	17 (18.3%)	7 (14.9%)	0.62
Atrial fibrillation, *n* (%)	31 (33.3%)	13 (27.7%)	0.50
Hypertension, *n* (%)	48 (51.6%)	27 (57.4%)	0.52
Diabetes mellitus, *n* (%)	6 (6.5%)	1 (2.1%)	0.27
ICA occlusion, *n* (%)	23 (24.7%)	16 (34.0%)	0.25
M1 occlusion, *n* (%)	50 (53.8%)	21 (44.7%)	0.31
M2 occlusion, *n* (%)	12 (12.9%)	5 (10.6%)	0.70
Tandem occlusion, *n* (%)	8 (8.6%)	5 (10.6%)	0.70
Baseline ASPECTS, median (IQR)	8 (7–8.5)	7 (7–8)	0.20
General anesthesia, *n* (%)	77 (82.8%)	39 (83.0%)	0.98
Time to start of treatment, minute, median (IQR)	255 (180–282)	228 (197–270)	1.00
Procedure time, minute, median (IQR)	32 (20–60)	33 (18–63)	0.55
Time to reperfusion, minute, median (IQR)	286 (222.5–336)	298 (238–342)	0.67
Stent retriever first, *n* (%)	31 (33.3%)	13 (27.7%)	0.50
Aspiration tubing first, *n* (%)	31 (33.3%)	15 (31.9%)	0.87
Combined technique first, *n* (%)	31 (33.3)	19 (40.4%)	0.41
Number of passages, median (IQR)	1 (1–2)	2 (1–3)	0.11
First-pass reperfusion, *n* (%)	47 (50.5%)	22 (46.8%)	0.68
Rescue therapy, *n* (%)	5 (5.4%)	5 (10.6%)	0.25
Successful reperfusion (mTICI 2b-3), *n* (%)	86 (92.5%)	38 (80.9%)	0.04^*^
Perforated complications, *n* (%)	5 (5.4%)	3 (6.4%)	0.81
Intracranial hemorrhage, *n* (%)	23 (24.7%)	21 (44.7%)	0.02^*^
Symptomatic intracranial hemorrhage, *n* (%)	2 (2.2%)	3 (6.4%)	0.20

*IQR, interquartile range*.

* *Indicates a significant statistical difference as P-value ≤ 0.05*.

**Table 3:** Logistic regression analysis of factors associated with a favorable outcome.

	**Unadjusted OR**	**95% CI**	***P*-value**	**Adjusted OR**	**95% CI**	***P-*value**
Age, per 1-year increase	0.97	0.94–1.00	0.03	0.96	0.92–1.00	0.05
Baseline NIHSS, per 1-point increase	0.82	0.74–0.90	0.00	0.82	0.74–0.92	0.00
Successful reperfusion (mTICI 2b-3)	0.34	0.12–0.99	0.05	4.98	0.82–30.34	0.08
Intracranial hemorrhage	2.46	1.17–5.17	0.02	0.29	0.10–0.81	0.02

### Prognostic Factors for Mortality

The differences between survivors and patients who died based on baseline NIHSS and the ICA occlusion rate are displayed in Table 4. Interestingly, further logistic regression analysis revealed that higher baseline NIHSS (OR, 1.22; 95% CI, 1.01–1.46; *P* = 0.04), unsuccessful revascularization (OR, 0.02; 95% CI, 0.00–0.58; *P* = 0.02), and rate of any ICH (OR, 0.12; 95% CI, 0.02–0.75; *P* = 0.02) were independently associated with mortality at 3 months, as shown in Table 5. When dichotomized, patients with baseline NIHSS > 14 had a higher mortality rate than those with baseline NIHSS ≤ 14 (18.8 vs. 4.3%; OR, 5.08; 95% CI, 1.47–17.49; *P* = 0.01).

**Table 4:** Factors associated with mortality.

**Patient characteristics** **(*n* = 140)**	**Survival group** **(*n* = 127)**	**Death group** **(*n* = 13)**	***P-*value**
Age, year, median (IQR)	66 (58–74)	65 (61.5–81)	0.18
Gender, male, *n* (%)	62 (48.8%)	5 (38.5%)	0.48
Baseline NIHSS, median (IQR)	13 (10–16)	16 (14–20.5)	0.01^*^
Previous stroke, *n* (%)	23 (18.1%)	1 (7.7%)	0.34
Atrial fibrillation, *n* (%)	40 (31.5%)	4 (30.8%)	0.96
Hypertension, *n* (%)	68 (53.5%)	7 (53.8%)	0.98
Diabetes mellitus, *n* (%)	6 (4.7%)	1 (7.7%)	0.64
ICA occlusion, *n* (%)	32 (25.2%)	7 (53.8%)	0.03^*^
M1 occlusion, *n* (%)	67 (52.8%)	4 (30.8%)	0.13
M2 occlusion, *n* (%)	16 (12.6%)	1 (7.7%)	0.61
Tandem occlusion, *n* (%)	12 (9.4%)	1 (7.7%)	0.84
Baseline ASPECTS, median (IQR)	8 (7–8)	8 (7–8.5)	0.66
General anesthesia, *n* (%)	104 (81.9%)	12 (92.3%)	0.34
Time to start of treatment, minute, median (IQR)	250 (190–278)	265 (182.5–272.5)	0.81
Procedure time, minute, median (IQR)	32 (20–60)	33 (15–63)	0.67
Time to reperfusion, minute, median (IQR)	290 (230.5–334)	298 (208.5–347.5)	0.68
Stent retriever first, *n* (%)	41 (32.3%)	3 (23.0%)	0.50
Aspiration tubing first, *n* (%)	41 (32.3%)	5 (38.5%)	0.65
Combined technique first, *n* (%)	45 (35.4%)	5 (38.5%)	0.83
Number of passages, median (IQR)	1 (1–2)	1 (1–2.5)	0.65
First-pass reperfusion, *n* (%)	61 (48.0%)	8 (61.5%)	0.35
Rescue therapy, *n* (%)	8 (6.3%)	2 (15.4%)	0.23
Successful reperfusion (mTICI 2b-3), *n* (%)	114 (89.8%)	10 (76.9%)	0.17
Perforated complications, *n* (%)	8 (6.3%)	0 (0%)	0.35
Intracranial hemorrhage, *n* (%)	38 (29.9%)	6 (46.2%)	0.23
Symptomatic intracranial hemorrhage, *n* (%)	4 (3.1%)	1 (7.7%)	0.40

*IQR, interquartile range*.

* *Indicates a significant statistical difference as P-value ≤ 0.05*.

**Table 5:** Logistic regression analysis of factors associated with mortality.

	**Unadjusted OR**	**95% CI**	***P-*value**	**Adjusted OR**	**95% CI**	***P-*value**
Baseline NIHSS, per 1-point increase	1.18	1.03–1.33	0.01	1.22	1.01–1.46	0.04
Internal carotid artery occlusion	0.29	0.09–0.92	0.04	15.95	0.32–798.39	0.17
Successful reperfusion (mTICI 2b-3)	2.63	0.64–10.80	0.18	0.02	0.00–0.58	0.02
Intracranial hemorrhage	0.50	0.16–1.58	0.24	0.12	0.02–0.75	0.02

### Subgroup Analysis

We found that the median mRS at 90 days was significantly better in the MCA occlusion group compared to the ICA plus M1 occlusion group [1 (IQR 0–3) vs. 2 (QR 1–4); *P* = 0.05] (Table 6). Median age, baseline ASPECTS, time to start of treatment, procedure time, time to reperfusion, successful reperfusion rate, and rate of any ICH did not show statistically significant differences. Only the median baseline NIHSS was found significantly lower in the MCA occlusion group when compared to the ICA plus M1 occlusion group [12 (IQR 10–15) vs. 14 (QR 12–18); *P* = 0.02] (Table 6).

**Table 6:** Subgroup analysis based on occlusion site.

**Patient characteristics** **(*n* = 140)**	**MCA occlusion group (*n* = 87)**	**ICA plus M1 occlusion group (*n* = 45)**	***P-*value**
Age, year, median (IQR)	66 (59–74)	65 (56–79)	0.10
Gender, male, *n* (%)	41 (47.1%)	26 (49.1%)	0.85
Previous stroke, *n* (%)	13 (14.9%)	5 (11.1%)	0.55
Atrial fibrillation, *n* (%)	28 (32.2%)	16 (35.6%)	0.70
Hypertension, *n* (%)	49 (56.3%)	20 (44.4%)	0.18
Diabetes mellitus, *n* (%)	5 (5.7%)	0 (0%)	0.10
Baseline NIHSS, median (IQR)	12 (10–15)	14 (12–18)	0.02^*^
Baseline ASPECTS, median (IQR)	7 (7–8)	7 (7–9)	0.85
General anesthesia, *n* (%)	70 (80.5%)	38 (84.4%)	0.58
Time to start of treatment, minute, mean (SD)	236.3 (64.7)	237.4 (59.6)	0.49
Procedure time, minute, mean (SD)	40.6 (32.4)	46.4 (31.7)	0.29
Time to reperfusion, minute, mean (SD)	278.1 (76.4)	283.8 (71.6)	0.69
Stent retriever first, *n* (%)	29 (33.3%)	9 (20.0%)	0.11
Aspiration tubing first, *n* (%)	27 (31.0%)	18 (40.0%)	0.30
Combined technique first, *n* (%)	31 (35.6%)	18 (40.0%)	0.62
Number of passages, median (IQR)	1 (1–2)	2 (1–3)	0.41
First-pass reperfusion, *n* (%)	42 (48.3%)	20 (44.4%)	0.68
Rescue therapy, *n* (%)	2 (2.3%)	4 (8.9%)	0.09
Successful reperfusion (mTICI 2b-3), *n* (%)	77 (88.5%)	39 (86.7%)	0.76
Perforated complications, *n* (%)	7 (8.0%)	1 (2.2%)	0.19
Any type of intracranial hemorrhage, *n* (%)	32 (36.8%)	11 (24.4%)	0.15
Symptomatic intracranial hemorrhage, *n* (%)	3 (3.4%)	2 (4.4%)	0.78
mRS at 90 days, median (IQR)	1 (0–3)	2 (1–4)	0.05^*^

*IQR, interquartile range; SD, standard deviation*.

* *Indicates a significant statistical difference as P-value ≤ 0.05*.

## Discussion

In this study, direct MT for AC-LVO within 4.5 h was associated with a high rate of successful reperfusion (124/140 patients, 88.6%) leading to a good outcome in 66.4% of patients (93/140) at 3 months. In comparison, the rates of successful reperfusion in recent clinical trials of direct MT were 79.4% in the DIRECT-MT trial, 88.5% in the DEVT trial, 90.1% in the SKIP trial, 78.7% in the MR CLEAN-NO IV trial, and 91% in the SWIFT-DIRECT trial (16–20).

Meanwhile, the rate of good outcomes of direct MT observed in our study (66.4%) was higher than in the DIRECT-MT (36.5%), DEVT (58%), SKIP (59.4%), MR CLEAN-NO IV (49.1%), and SWIFT-DIRECT (58%) trials (16–20). Several factors may explain this difference. First, the stroke severity was milder in our group of patients with a median baseline NIHSS of 13.5 (IQR, 11–16). In contrast, the median baseline NIHSS was 17 (IQR, 12–21) in the DIRECT-MT trial (16), 16 (IQR, 12–20) in the DEVT trial (17), 19 (IQR, 12–23) in the SKIP trial (18), 16 (IQR, 10–20) in the MR CLEAN-NO IV trial (19), and 17 (IQR, 13–20) in the SWIFT-DIRECT trial (20). Lower baseline NIHSS was confirmed as an independent factor associated with good outcomes at 3 months in our study (OR, 0.82; 95% CI, 0.74–0.92; *P* = 0.00) and the study of Yoon et al. (OR, 0.89; 95% CI, 0.84–0.94; *P* < 0.001) (24). Second, the rate of any ICH (31.4%) in our cohort was lower than in the DIRECT-MT trial (37.6%), SKIP trial (33.7%), and MR CLEAN-NO IV trial (35.9%) (16, 18, 19). As shown in Table 3, the presence of any ICH is an independent predictor of a poor outcome at 3 months (OR, 0.29; 95% CI, 0.10–0.81; *P* = 0.02). This finding concurs with the association between parenchymal hemorrhage and poor clinical outcomes reported in Yoon et al.'s study (OR, 0.15; 95% CI, 0.05–0.46; *P* = 0.00) (24). Third, the patients in our cohort were younger (median age 65.5, IQR from 59 to 76.5) than those in the DIRECT-MT trial (median age 69, IQR from 61 to 76), DEVT trial (median age 70, IQR from 60 to 77), SKIP trial (median age 74, IQR from 67 to 80), MR CLEAN-NO IV trial (median age 72, IQR from 62 to 80), and SWIFT-DIRECT trial (median age 73, IQR from 64 to 81) (16–20). The relationship we observed in our study between younger age and favorable clinical outcome (OR, 0.96; 95% CI 0.92–1.00; *P* = 0.05) was also found in the study of Yoon et al. (OR, 0.96; 95% CI, 0.94–0.99; *P* = 0.00) (24). While successful reperfusion was also noted by Yoon et al. as an independent predictor for a good outcome (OR, 4.66; 95% CI, 2.24–9.69; *P* < 0.001) (24), we observed this relationship only in univariate (OR, 0.34; 95% CI, 0.12–0.99; *P* = 0.05) but not in multivariate analysis (OR, 4.98; 95% CI, 0.82–30.34; *P* = 0.08). This may be explained by our relatively small sample size (*n* = 140) compared to that of Yoon et al. (*n* = 335) (24).

Regarding the effectiveness of direct MT compared to the combination of MT and IVT, a comparison has been performed based on preliminary results of our interventional trial from April 2020 to March 2022 (Supplementary Table 7). The successful reperfusion (mTICI 2b-3) rate at the end of the procedure in the direct MT group was 91.1% (41/45 patients), which was similar to the successful reperfusion rate of the combination therapy group (91.1%, 41/45 patients) (Supplementary Table 7). However, the favorable clinical outcome rate achieved in the direct MT group was slightly higher than in the combination therapy group (66.7 vs. 64.4%; *P* = 0.83) (Supplementary Table 7). This corresponds well with Yoon et al. (24) and confirms the previous findings of Raoult et al. (25) that pretreatment with IVT is not an independent prognostic factor for good outcomes after MT. As proposed by Yoon et al. (24), the evidence we found points to three independent prognostic factors after MT: younger age, lower baseline NIHSS, and absence of ICH post-treatment. First, the median age of patients in the direct MT group (64; IQR, 58.5–72.5) was lower than patients treated with combined therapy (68; IQR, 60–74) (Supplementary Table 7). Second, the baseline NIHSS was lower for patients in the direct MT group (12; IQR, 10–14) when compared with patients treated with the combined therapy (13; IQR, 11–16) (Supplementary Table 7). Finally, any type of ICH rate of patients in the direct MT group was also lower compared to patients treated with the combined therapy (42.2 vs. 46.7%) (Supplementary Table 7).

We observed a mortality rate of 9.3% at 3 months post-treatment. This rate was lower than the reported mortality rate of the direct MT group in the SWIFT-DIRECT trial (11%) (20), the DEVT trial (17.2%) (17), the DIRECT-MT trial (17.7%) (16), and the MR CLEAN-NO IV trial (20.5%) (19), but higher than the SKIP trial (7.9%) (18). To identify predictive factors of mortality at 3 months, we found an association between high baseline NIHSS (OR, 1.22; 95% CI, 1.01–1.46; *P* = 0.04), unsuccessful reperfusion (OR, 0.02; 95% CI, 0.00–0.58; *P* = 0.02), the presence of any ICH (OR, 0.12; 95% CI, 0.02–0.75; *P* = 0.02) and mortality (Table 5). Our study found a lower baseline NIHSS, a higher reperfusion rate, and a lower ICH rate than the DIRECT-MT trial (16), and the MR CLEAN-NO IV trial (19). The correlation between unsuccessful reperfusion, presence of any ICH, and mortality outcome was also identified in the study of Yoon et al. (24).

Surprisingly, in subgroup analysis, the clinical outcome at 90 days was significantly better in the MCA occlusion group (median mRS 1, IQR 0–3) compared to the ICA plus M1 occlusion group (median mRS 2, IQR 1–4) (*P* = 0.05; Table 6). There are several possible explanations for this result. First, the median baseline NIHSS was significantly lower in the MCA occlusion group when compared to the ICA plus M1 occlusion group (Table 6). Second, the mean duration time from the femoral puncture to reperfusion was ~6 min shorter in the MCA occlusion group compared with the ICA plus M1 occlusion group. And third, the first-pass reperfusion rate was higher in the MCA occlusion group than in the ICA plus M1 occlusion group (Table 6). The combination with IVT is known to be associated with both benefits and risks for subsequent thrombectomy (26). On the one hand, IVT may soften the thrombus and facilitate the thrombectomy procedure with fewer passages, thus, reducing the procedure time (27–30). This benefit was also observed in the preliminary results of the interventional trial conducted at our center. The pre-treatment with IVT increased the rate of first-pass reperfusion and this benefit appeared to be higher in the ICA occlusion group (increased by 8.9%) compared to the MCA occlusion group (increased by 4.6%) (Supplementary Table 7). However, on the other hand, IVT may lead to a higher rate of distal thrombus migration (8.9 vs. 0%, *P* = 0.04; Supplementary Table 7). The migration of the thrombus in the proximal MCA (M1, M2) to more distant segments (M3, M4) may make subsequent thrombectomy more difficult or impossible (26). Our finding correlates with the results of the SKIP trial where direct MT was more favorable in the case of M1 occlusion compared to ICA occlusion (18). Subgroup analysis of the occlusion site was not reported in other RCTs (16, 17, 19, 20). A pooled individual patient data meta-analysis from recent RCTs is required to explore this finding (16–20).

Our study has three main limitations. First, the limited number of cases in a single-center study may overestimate outcome differences. Second, the study employs a heterogeneity of protocol (retrospective and prospective combination design) which might cause selection bias. Finally, several other confounding factors that may influence the final clinical outcome such as other diseases (cardiac failure, brain atrophy, leukoaraiosis), biomarkers, the clot characteristics, the clot length/volume, and collateral circulation were not evaluated in our study.

## Conclusions

The present study suggests that direct mechanical thrombectomy may be a sufficient option in younger patients (≤70) presenting proximal middle artery occlusion within 4.5 h with low baseline NIHSS (≤14). A pooled individual patient meta-analysis from recent RCTs is required to validate these results.

## Data Availability Statement

The raw data supporting the conclusions of this article will be made available by the authors upon reasonable request.

## Ethics Statement

The studies involving human participants were reviewed and approved by the Independent Ethics Committee of Vietnam's Ministry of Health. Written informed consent from the patients/participants or patients/participants' legal guardian/next of kin was not required to participate in this study in accordance with the national legislation and the institutional requirements.

## Author Contributions

HAN, DLV, and QAN substantially contributed to the acquisition, analysis, and data interpretation. HAN and DLV prepared, drafted, and revised the manuscript for important intellectual content. All author gave final approval of the version to be published and agreed to be accountable for all aspects of the work, ensuring that questions related to the accuracy, or integrity of any part of the work are appropriately investigated and resolved.

## Conflict of Interest

The authors declare that the research was conducted in the absence of any commercial or financial relationships that could be construed as a potential conflict of interest.

## Publisher's Note

All claims expressed in this article are solely those of the authors and do not necessarily represent those of their affiliated organizations, or those of the publisher, the editors and the reviewers. Any product that may be evaluated in this article, or claim that may be made by its manufacturer, is not guaranteed or endorsed by the publisher.
