# Supplementary material for: Predictive Factors for Clinical Outcome After Direct Mechanical Thrombectomy for Anterior Circulation Large Vessel Occlusion Within 4.5 h
Source: Front Neurol. 2022 Jun 30;13:895182. doi: 10.3389/fneur.2022.895182 (PMC9280660; doi:10.3389/fneur.2022.895182)
Supplement: Supplementary file 1 [file Data_Sheet_1.PDF]

Table 7. Preliminary results of the interventional trial comparing direct thrombectomy with combination therapy from April 2020 to March 2022

	Direct MT (n = 45)	IVT plus MT (n = 45)	*P*
**Demographic characteristics**			
Age, median (IQR)	64 (58.5–72.5)	68 (IQR, 60–74)	0.70†
Men, n (%)	26 (57.8)	32 (71.1)	0.19§
**Risk factor**			
Previous ischemic stroke, n (%)	10 (22.2)	8 (17.8)	0.60§
Atrial fibrillation, n (%)	15 (33.3)	18 (40.0)	0.51§
Diabetes mellitus, n (%)	5 (11.1)	4 (8.9)	0.73§
Hypertension, n (%)	25 (55.6)	23 (51.1)	0.67§
Smoking, n (%)	4 (8.9)	2 (4.4)	0.40§
**Pre-mRS,** median (IQR)	0 (0–0)	0 (0–0)	0.31§
**Stroke etiology**			
Cardioembolism, n (%)	18 (40.0)	26 (57.8)	0.09§
Large-artery atherosclerosis, n (%)	21 (46.7)	14 (31.1)	0.13§
**Clinical examination**			
Time from onset to admission, minutes, median (IQR)	167 (102–219.5)	145 (105–194.5)	0.08†
NIHSS score, median (IQR)	12 (10–14)	13 (11–16)	0.24§

Initial glucose level, mg/dL, mean (SD)	6.9±1.8	7.0±2.1	0.67†
**Imaging characteristics**			
Baseline ASPECTS, median (IQR)	7 (7–8)	7 (7–8)	0.80§
ICA occlusion, n (%)<br>M1 occlusion, n (%)<br>M2 occlusion, n (%)	13 (28.9)<br>28 (62.2)<br>4 (8.9)	19 (42.2)<br>21 (46.7)<br>5 (11.1)	0.19§<br>0.14§<br>0.73§
**Endovascular therapy characteristics**			
Time from admission to start of alteplase, minutes, median (IQR)	NA	39 (23.5-49.8)	NA
Time from admission to groin puncture, minutes, median (IQR)	63 (50.3-86.0)	74 (61.8-88.0)	0.26†
Time from groin puncture to reperfusion, minutes, median (IQR)	38 (23-73.5)	36 (30-62.5)	0.52†
General anesthesia, n (%)	38 (84.4)	39 (86.7)	0.77§
**First thrombectomy device**<br>Aspiration tubing, n (%)<br>Stent retriever, n (%)<br>Combination of aspiration tubing and stent retriever, n (%)	 12 (26.7)<br>5 (11.1)<br>28 (62.2)	 4 (8.9)<br>7 (15.6)<br>33 (73.3)	 0.03§*<br>0.54§<br>0.26§
Median number of passages (IQR)	2 (1-2)	1 (1-2)	0.35§

**Rescue techniques**			0.56§
Balloon angioplasty alone, n (%)	5 (11.1)	4 (8.9)	
Carotid stenting, n (%)	2 (4.4)	1 (2.2)	
Intracranial stenting, n (%)	1 (2.2)	1 (2.2)	
Periprocedural complication, n (%)	3 (6.7)	3 (6.7)	0.98§
Thrombus migration, n (%)	0 (0)	4 (8.9)	0.04§*
**Outcome characteristics**			
Successful reperfusion (mTICI 2b-3), n (%)	41 (91.1)	41 (91.1)	1.00§
First-pass successful reperfusion (mTICI 2b-3), n (%)	22 (48.9)	24 (53.3)	0.68§
ICA occlusion group, n (%)	5/13 (38.5)	9/19 (47.4)	
MCA occlusion group, n (%)	17/32 (53.1)	15/26 (57.7)	
Any type of ICH, n (%)	19 (42.2)	21 (46.7)	0.67§
Symptomatic ICH, n (%)	0 (0)	1 (2.2)	0.32§
Good outcome (mRS 0-2), n (%)	30 (66.7)	29 (64.4)	0.83§
mRS at 90 days, median (IQR)	2 (1-3)	2 (1-3)	0.76§
Mortality, n (%)	2 (4.4)	2 (4.4)	1.00§

MT, mechanical thrombectomy; IVT, intravenous thrombolysis; IQR, interquartile range; NA, not applicable; †*P* was calculated using the Student's t-test; §*P* was calculated using the Mann–Whitney U Test; **P* ≤ 0.05
